# From signals to solutions: stress-induced leaf senescence and synthetic biology and AI approaches for crop resilience

**DOI:** 10.1186/s43897-026-00236-9

**Published:** 2026-06-11

**Authors:** Shu-Ning Ren, Chen-Yu Zhu, Yu-Qiong Wang, Tian Bu, Zhonghai Li, Weilun Yin, Xinli Xia, Hou-Ling Wang

**Affiliations:** State Key Laboratory of Tree Genetics and Breeding, School of Biological Sciences and Technology, Beijing Forestry University, Beijing, 100083 P. R. China

**Keywords:** Leaf senescence, Abiotic stress, Senescence-associated genes, Synthetic biology, Breeding

## Abstract

The quality and quantity of plant traits are critically linked to the coordinated onset of leaf senescence. However, both external environmental factors and internal hormones may accelerate leaf senescence process, resulting in various physiological changes, including chlorophyll degradation, anthocyanin biosynthesis, nutrient recycling, and the activation of senescence-associated genes (*SAGs*). A comprehensive understanding of the signaling pathways involved in stress-induced leaf senescence is essential for plant breeding aimed at enhancing resistance and productivity. This review provides an extensive overview of the signaling mechanisms associated with leaf senescence triggered by abiotic and biotic stresses, including abscisic acid (ABA), darkness, nitrogen deficiency, carbon deficiency, and pathogen attack. Additionally, we discuss strategies to improve stress tolerance, yield, and quality through innovative synthetic biology approaches. Furthermore, we explore the potential applications of machine learning (ML) and deep learning (DL) in the context of senescence- and stress-related plant breeding.

## Introduction

Leaves serve as vital photosynthetic organs that capture energy and synthesize nutrients. Leaf senescence is characterized as the final stage of leaf development, representing an age-dependent degradation process that affects cells, tissues, organs, and entire organisms in plants. A prominent visual indicator of leaf senescence is leaf yellowing, which is closely linked to the degradation of the green pigment chlorophyll. In numerous plant species, leaf yellowing occurs in a coordinated manner, typically initiating at the leaf tip and edges before progressing toward the base and petiole (Avila et al. 2014). Leaf senescence is a vital biological and developmental process that influences leaves and the whole plant (Guo et al. 2021, 2025; Wang et al. 2023a, b). This process is essential for optimizing production and ensuring the survival of successive generations of plants (Bleecker et al. 1997; Woo et al. 2019). For example, plants such as soybeans, rice, and corn undergo senescence, which ultimately results in the death of the entire organism, including leaves and roots (Lim et al. 2007).

**Fig. 1:**
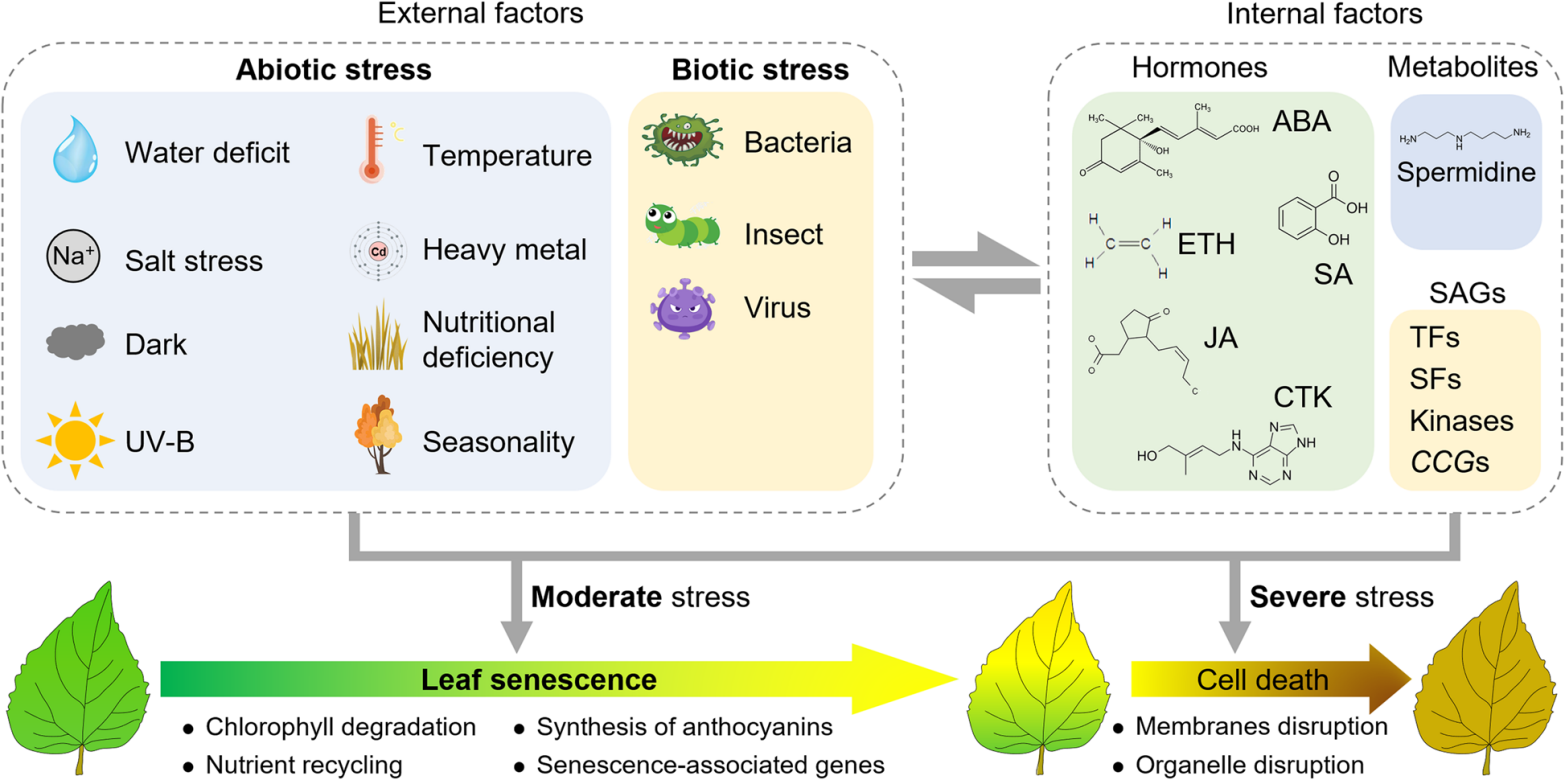
A model for stress-induced leaf senescence. External and internal factors are integrated for onset of leaf senescence process. Moderate stress drives normal leaf senescence while severe stress directly leads to cell death

During the process of senescence, leaves engage in the redistribution of nutrients to developing tissues or storage organs while simultaneously undergoing the degradation of intracellular organelles and the breakdown of macromolecules. Consequently, leaf senescence plays a pivotal role in the recycling of nutrients within plants (Lim et al. 2007). In annual plants, the degraded biomacromolecules and disassembled nutrients are allocated to developing organs, such as seeds or other essential parts, thereby facilitating the continuation of plant offspring. In perennial plants, such as temperate deciduous trees, the aesthetic phenomenon of leaf abscission occurs each autumn, reflecting the characteristics of senescence (Woo et al. 2019). In these species, nutrients are reallocated to the stems or roots in preparation for the development of vegetative organs, such as new leaves, in the subsequent growing season. Senescence is influenced by both internal factors and external environmental factors. Various external factors, including biotic stressors, significantly impact the process of leaf senescence. Abiotic stressors, such as temperature fluctuations, water deficits, salt stress, heavy metal exposure, nutritional deficiencies, seasonal changes, and intense light, along with biotic stressors, including pathogens, insects and viruses, often lead to visible symptoms of senescence, such as leaf yellowing. The interplay of diverse internal and external cues modulates the expression of multiple senescence-associated genes (*SAGs*) within the signaling networks of plant leaves. The activation of ABA and salicylic acid (SA) signaling, the synergistic and specific regulation of ethylene (ETH) and jasmonic acid (JA) coupled with the degradation of cytokinin (CTK), orchestrates plant senescence programs, thereby laying the molecular and physiological foundation for subsequent nutrient remobilization and plant growth regulation (Fig. 1).

## Leaf senescence induced by abiotic stress

Stress-signaling pathways are intricately associated with the regulatory processes of leaf senescence, as numerous pathways modulate the expression of *SAGs*. As the main hormone regulating the senescence of plant leaves, ABA plays a crucial role in abiotic stress and the senescence of leaves. In higher plants, ABA plays a pivotal role in mediating stress responses and regulating multiple developmental processes, including seed maturation and dormancy, organ abscission, and leaf senescence (Cutler et al. 2010; Chen et al. 2020a, b). Notably, ABA levels are tightly regulated by the dynamic equilibrium between its biosynthesis and catabolism (Nambara et al. 2005), and a marked elevation in ABA concentrations is a characteristic feature of leaves undergoing senescence (Lim et al. 2007; Mao et al. 2017; Zhang et al. 2021), implying its active involvement in senescence progression.

**Fig. 2:**
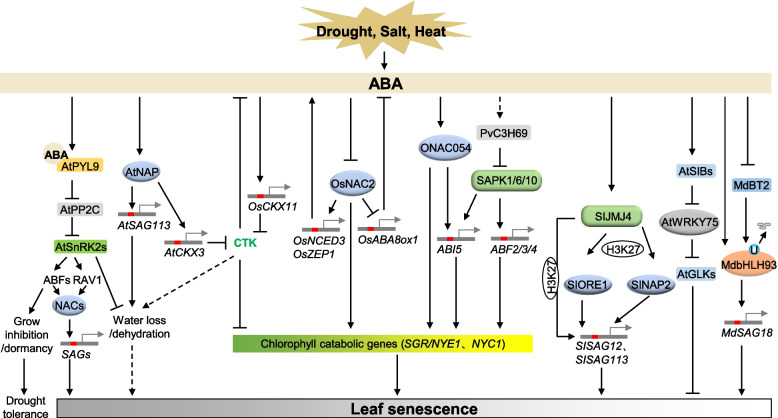
Regulatory network integrated by abiotic stress-induced hormone ABA. ABA: abscisic acid; CTK: Cytokinin; ROS: Reactive oxygen species

During leaf senescence, ABA exerts its regulatory effects through complex signaling networks and interconnected pathways that dynamically modulate the senescence state. Extensive studies have elucidated the ABA biosynthetic pathways and downstream signaling mechanisms, with a particular focus on upstream transcriptional networks governing ABA levels during senescence and their consequent impacts on the senescence process. Mechanistically, ABA promotes leaf senescence and abscission primarily by inducing the expression of *SAGs*, such as *NYC1* (NON-YELLOW COLORING 1) (Kusaba et al. 2007), *SGR1/NYE1* (STAY-GREEN 1, also known as NONYELLOWING 1) (Park et al. 2007; Ren et al. 2007), *PPH* (PHEOPHYTINASE) (Schelbert et al. 2009), *NACs* (Kai et al. 2019), *WRKY75* (WRKY DNA-BINDING PROTEIN 75) (Zhang et al. 2022b), these genes collectively drive senescence-associated physiological changes (Fig. 2).

Furthermore, ABA signaling is closely linked to the regulation of *NAC* transcription factors, a family of key senescence regulators. Following ABA treatment, the expression of several *NAC* members including *SNAC-As* (Takasaki et al. 2015), *VNI2* (VND-interacting 2) (Yang et al. 2011), *ORE1* (ORESARA 1) (Kim et al. 2011), and *OsNAP (NAC-like, activated by AP3/PI)* (Liang et al. 2014), is significantly upregulated, though the underlying molecular mechanisms remain to be fully characterized. Specifically, AtNAP, a well-characterized NAC transcription factor, can directly bind to the promoter of AAO3 (ALDEHYDE OXIDASE 3), a key enzyme in ABA biosynthesis, thereby enhancing the transcription of chlorophyll-degrading genes and forming a regulatory loop that amplifies ABA-mediated senescence signals (Yang et al. 2014).

In the process of plant senescence, hormone regulation is not the ‘single action’ of ABA, but a complex network in which multiple endogenous hormones respond in coordination and participate together. Besides the fact that ABA regulates senescence through its synthesis and signaling pathways, other hormones such as SA, jasmonic acid (JA), and ethylene will also rapidly adjust their metabolism and signal transduction during the senescence process. Moreover, peptide hormones are involved in stress-induced leaf senescence. Small secreted peptide CLE14 (CLAVATA3/ESR-RELATED 14) is a novel suppressor of leaf senescence, and its expression is induced by aging, ABA, and stress signals to delay both natural and stress-induced senescence. The function of CLE14 is mediated by its activation of the transcription factor JUB1 (JUNGBRUNNEN1), which enhances the expression of reactive oxygen species (ROS) scavenging genes and reduces oxidative stress, thereby establishing a regulatory peptide pathway that fine-tunes leaf senescence timing (Zhang et al. 2022c, d).

Specifically for JA, its regulation of aging is reflected not only in the basic aging process but also in the coordination of aging under adverse conditions: During the natural senescence of leaves, enzyme catalytic genes of JA-related biosynthetic pathways are differentially activated (He et al. 2022). For example, the transcription factor BpTCP19 (TEOSINTE BRANCHED1/CYCLOIDEA/PROLIFERATING CELL FACTOR 19), acts as a central negative regulator of leaf senescence, can mediate histone deacetylation. Reduce the binding of inhibitory factors *JAZ4* (*JASMONATE-ZIM DOMAIN 4*) and *JAZ8* (*JASMONATE-ZIM DOMAIN 8*) in the JA signaling pathway to WRKY57 (WRKY DNA-BINDING PROTEIN 57), thereby inhibiting the senescence process (Wang et al. 2024). Under the adverse condition of cold exposure, the regulation of JA shifts to a ‘stress-senescence’ synergistic response. At this time, genes such as *OsLOX2* (*LIPOXYGENASE 2*), *OsAOC* (*ALLENE OXIDE CYCLASE*), *OsAOS1* (*ALLENE OXIDE SYNTHASE 1*) and *OsAOS2* (*ALLENE OXIDE SYNTHASE 2*) related to JA biosynthesis in rice will be significantly upregulated, promoting the initiation of the coordination mechanism between stress adaptation and senescence in leaves to cope with harsh environments (Hu et al. 2017). In the ethylene regulatory pathway, its promoting effect on leaf senescence depends on the key transcription factor *EIN3* (ETHYLENE-INSENSITIVE3)*.* As an aging-related gene with functional activity, *EIN3* can participate in and regulate the chlorophyll degradation process in naturally senescent leaves by directly binding to the promoter region of CCGs (chlorophyll catabolism genes), thereby promoting senescence progression (Yang et al. 2020).

### The impact of water conditions on leaf senescence

Under adverse conditions such as drought or waterlogging, plants have evolved premature leaf senescence as an adaptive strategy to reallocate nutrients from senescing leaves to vital organs, thereby sustaining overall plant growth and survival (Guiboileau et al. 2010; Woo et al. 2019). This phenomenon is particularly pronounced under water deficiency, where ABA emerges as a central regulator orchestrating both stress adaptation and senescence progression. In water-deficient environments, elevated ABA biosynthesis triggers rapid stomatal closure, a key physiological response that minimizes transpiration water loss and enhances drought tolerance (Yamaguchi-Shinozaki et al. 2006). Concomitantly, accumulating ABA serves as a critical signaling molecule to initiate leaf senescence, linking stress perception to the activation of senescence programs. The coordination of these processes relies on a suite of drought-responsive genes, with those involved in ABA biosynthesis and stress-protective pathways being indispensable for plant adaptation to water scarcity (Furihata et al. 2006; Urano et al. 2009).

Mechanistically, the SnRK2 (SNF1-related kinase 2) family plays a pivotal role in transducing ABA signals and these kinases phosphorylate downstream substrates, including ABA-responsive element binding factors (*ABFs*) and other transcription factors, to regulate the expression of ABA-responsive genes involved in stress adaptation and senescence (Zhao et al. 2018) (Fig. 2). ABA perception by its receptors, the PYL (Pyrabactin Resistance 1-Like) proteins, further modulates SnRK2 activity under osmotic stress, with evidence indicating that *PYL*-mediated regulation can function independently of core ABA signaling modules (Asad et al. 2019). Notably, specific *PYL* members are directly implicated in senescence regulation: transgenic *Arabidopsis* expressing *PYL9* under the drought-inducible *RD29A* promoter (*pRD29A::PYL9*) exhibits both enhanced drought resistance and accelerated drought-induced leaf senescence, highlighting *PYL9* as a molecular link between ABA signaling, stress tolerance, and senescence initiation. Furthermore, ABA promotes leaf senescence through a pathway distinct from ethylene signaling: activated SnRK2s phosphorylate not only ABFs (ABA RESPONSIVE ELEMENT-BINDING FACTORs) but also RAV1 (Related to ABA-Insensitive 3/VP1) transcription factors, thereby driving the expression of *SAGs* independently of ethylene-mediated pathways (Zhao et al. 2016). Collectively, these findings underscore ABA as a multifunctional regulator that integrates drought stress responses with leaf senescence programs through a complex signaling network involving PYL receptors, SnRK2s, and downstream transcription factors. *In Arabidopsis thaliana*, AtNAP transcription factor directly activates *AtCKX3* (*CYTOKININ OXIDASE/DEHYDROGENASE 3*) to degrade cytokinins, leading to water loss and senescence, thereby integrating the ABA and CTK pathways to regulate leaf senescence (Zhang et al. 2012; Hu et al. 2021) (Fig. 2).

Over the past century, the frequency of global farmland waterlogging events has increased markedly, and this stress has caused substantial crop yield losses. The physiological damage caused by soil waterlogging to crops follows a clear cascade. First, waterlogging drastically reduces the oxygen exchange rate between plant roots and the soil (Armstrong et al. 1980), impairing root carbon sequestration, and it further induces stomatal closure in leaves to limit internal CO₂ levels and carbon fixation (Striker et al. 2005), while decreasing root hydraulic conductivity. These root-derived impairments subsequently affect aboveground leaf function, leading to a significant reduction in photosynthetic rate and ultimately grain yield loss (Araki et al. 2012). Under prolonged water immersion, plants also undergo a critical metabolic shift: root respiration transitions from aerobic respiration to anaerobic fermentation, and ethanol produced in roots is transported to leaves. At this stage, leaves compensate for root oxidative metabolism to sustain the plant’s overall carbon metabolism (Ferner et al. 2012).

Notably, the damage of waterlogging to crops is not limited to physiological phenotypes, its regulatory mechanisms can be traced to the molecular level, with microRNAs (miRNAs) emerging as core regulators of plant responses to this abiotic stress. Specifically, waterlogging-induced hypoxia modulates the expression of specific miRNAs, which play a pivotal role in mediating the stress-senescence cascade (Mishra et al. 2022). For instance, in maize, most target genes of hypoxia-induced miRNAs contain key cis-acting elements related to anaerobic response or hormone induction, and these miRNAs ultimately mediate maize’s adaptive response to waterlogging by regulating the expression of these specific target genes (Zhang et al. 2008). In *A. thaliana*, functional characterization of miR775 further revealed its regulatory role. The miR775 perturbation (via overexpression or inhibition) leads to significant changes in the expression of *SAGs* (*SAG12*, *SAG29*, *ORE1*), ethylene signaling genes (*EIN2*, *EIN3*), and the ABA biosynthetic gene *NCED3 (NINE-CIS-EPOXYCAROTENOID DIOXYGENASE 3). The miR775-GALT9 (GALACTOSYLTRANSFERASE 9)* module, in particular, is involved in post-flooding plant recovery, and its regulatory function relies on the integration of ethylene signaling and ABA biosynthesis pathways, directly linking miRNA-mediated molecular regulation to the physiological processes of stress adaptation and senescence (Mishra et al. 2022).

### Dark-induced leaf senescence

Darkness and light perception via plant pigments are pivotal regulators of leaf senescence, with their effects mediated by a coordinated network of molecular pathways and hormonal signaling cascades (Niu et al. 2012; Li et al. 2013; Durgud et al. 2018; Ding et al. 2022).

**Fig. 3:**
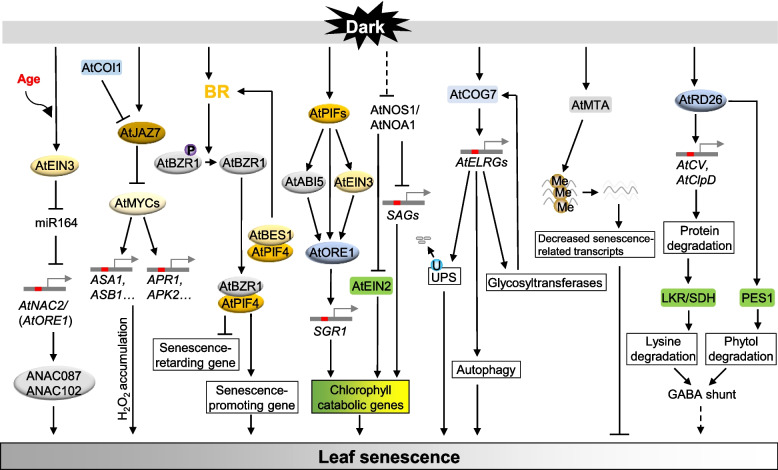
Dark-induced leaf senescence signaling and regulatory network. BR, brassinosteriods

At the molecular level, darkness strongly upregulates the expression of *SAGs*, with key regulators identified across multiple regulatory layers. For instance, *RD26* (*RESPONSIVE TO DESICCATION 26*), an ATAF (*A. thaliana* Activation Factor) subfamily transcription factor, acts as a positive regulator of dark-induced chlorophyll degradation in *A. thaliana*, and orchestrates metabolic reprogramming during senescence by modulating gene expression throughout the cellular degradation hierarchy (Kamranfar et al. 2018). Plant *PIGMENT INTERACTION FACTORS* (*PIFs*) form another core transcriptional module for dark-induced senescence: they establish a potential link to energy deprivation signaling pathways, and the complex, initially photo-reversible induction of this process is tightly tied to a *PIF*-dependent feedforward regulatory loop (Leivar et al. 2011; Cho et al. 2015; Liebsch et al. 2016; Sakuraba et al. 2020) (Fig. 3). Epigenetic regulation further contributes beyond transcription factors. Under dark conditions, N^6^-methyladenosine (m^6^A) synthesis increases in *A. thaliana*, gradually destabilizing senescence-related transcripts and inhibiting premature leaf senescence (Sheikh et al. 2024). Notably, the *COG* (*conserved oligomeric Golgi*) complex also supports dark stress resistance, and its mutations accelerate carbon deprivation and senescence under darkness (Choi et al. 2023).

Previous studies have highlighted the crucial role of transcription factors and epigenetic regulation in leaf senescence, as these regulatory layers precisely regulate the expression of *SAG*s and coordinate the initiation time of senescence. As a supplement to intracellular regulatory mechanisms, plant hormones, especially brassinosteroids (BR), JA, and ethylene act as key systemic signals, integrating dark processing signals into the senescence program, forming a multi-level regulatory network of dark-induced leaf senescence.

The plant hormones BR, JA, and ethylene play significant roles in the regulation of leaf senescence induced by darkness. BR plays a significant role in regulating dark-induced leaf senescence, and within the BR-mediated transcriptional regulatory network, *BES1* (*BR-INSENSITIVE-EMS-SUPPRESSOR1*) and *BZR1* (*BRASSINAZOLE-RESISTANT1*) serve as core transcription factors. They are mainly strictly regulated by the BR signal cascade through phosphorylation, which mediates crosstalk and regulatory connections across various pathways by regulating differentiated physiological processes like plant growth and development (Oh et al. 2014; Shi et al. 2022) (Fig. 3). Specifically, under dark conditions, BR synthesis is triggered, which subsequently activates *BZR1*, and this activation facilitates *BZR1* binding to the promoter of *AIF2* (*ATBS1-INTERACTING FACTOR 2*) in a darkness-dependent manner, leading to reduced *AIF2* transcription and thereby accelerating dark-induced leaf senescence (Kim et al. 2020). Notably, *AIF2* is a non-DNA-binding basic helix-loop-helix (bHLH) transcription factor that functions to delay both dark-induced and BR-induced leaf senescence in *A. thaliana*. Moreover, in apple plants, ABA promotes leaf senescence via the transcription factor MdbHLH93, which directly activates a senescence gene *MdSAG18*, while another ABA-responsive protein, MdBT2 (BTB and TAZ domain protein 2), counteracts senescence by inducing MdbHLH93 ubiquitination and degradation (An et al. 2019) (Fig. 2).

In addition to BR, which regulates dark-induced leaf senescence via modules such as *BZR1*-*AIF2*-JA and ethylene serve as core hormones coordinating this process, with JA exerting its function through distinct, species-specific transcriptional regulatory modules. In tomato, JA upregulates *SlMYC2* (*MYC transcription factor 2*), and *SlWRKY37* (*WRKY DNA-BINDING PROTEIN 37*), a downstream target of *SlMYC2*-subsequently enhances the expression of *SAGs* including *SlWRKY53* (*WRKY DNA-BINDING PROTEIN 53*) and *SlSGR1*, thereby promoting dark-induced senescence (Wang et al. 2022a, b). In birch, the transcription factor *BpTCP19* is downregulated under both dark conditions and methyl jasmonate (MeJA) treatment, and overexpression of *BpTCP19* delays senescence by reducing leaf sensitivity to JA and darkness (Wang et al. 2024). In *A. thaliana*, the JA signaling repressor *JAZ7* (*Jasmonate ZIM‑domain protein 7*) accumulates in the dark; mutations in *JAZ7* partially relieve its inhibitory effect on MYC2, MYC3 (MYC transcription factor 3), and MYC4, allowing these transcription factors to bind to the promoters of downstream genes and upregulate JA-related and senescence-promoting targets (Yu et al. 2016).

Beyond JA, ethylene regulates dark-induced leaf senescence through a suite of signaling components with divergent roles. Key ethylene signaling factors such as *EIN2* (*ETHYLENE-INSENSITIVE2*) (Alonso et al. 1999) and *EIN3* (*ETHYLENE-INSENSITIVE3*) (Qiu et al. 2015) primarily function as positive regulators of leaf senescence: they mediate ethylene-dependent signaling pathways and stress responses to induce the expression of *SAGs*, promote oxidative reactions in leaf cells, accelerate chlorophyll degradation, and activate downstream senescence regulators (e.g. NYEs, OREs), collectively driving dark-induced senescence (Kim et al. 2018). Conversely, factors like *SUB1A* (*SUBMERGENCE 1A*) act as negative regulators of senescence (Fukao et al. 2012). *SUB1A* modulates plant responses to JA and SA, inhibits the degradation of chlorophyll and carbohydrates, reduces the accumulation of senescence-related mRNA, and enhances both recovery from dark stress and tolerance to multiple adverse conditions. Notably, JA and ethylene often act synergistically to regulate senescence, a process inherently driving the degradation of leaf cellular macromolecules (e.g. proteins, nucleic acids), which is further exacerbated under dark conditions. Even senescent leaves maintained under light encounter subsequent oxidative stress due to reduced chlorophyll levels (Buchanan et al. 2005), highlighting how these hormones integrate environmental cues such as darkness and cellular physiological changes to fine-tune the senescence process.

### Nitrogen and carbon starvation induced leaf senescence

Nitrogen and carbon nutrients serve as significant determinants in the initiation of leaf senescence. Nitrogen, recognized as one of the most prevalent macronutrients, acts as a limiting factor in plant growth and development (Liu et al. 2017). A deficiency in nitrogen can expedite the senescence of plant leaves, and numerous regulatory factors are implicated in the senescence process that is influenced by nitrogen deficiency.

**Fig. 4:**
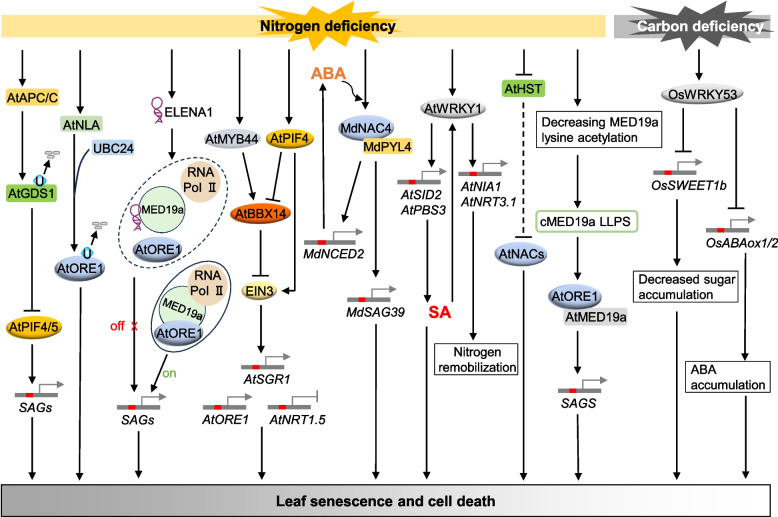
Nitrogen and carbon deficiency induced leaf senescence signaling and regulatory network

Nitrogen deficiency is a major abiotic stress triggering leaf senescence, with its regulatory network centered on key transcription factors and post-translational modifiers, while extensively cross-talking with hormone signaling pathways. The *NAC* transcription factor *ORE1* acts as a core hub in nutrients related leaf senescence. MED19a (MEDIATOR SUBUNIT 19a) binds the promoter of *ORE1* to activate nitrogen deficiency-induced senescence, and this activation is modulated by MED19 phase separation under nitrogen starvation (Cheng et al. 2022) (Fig. 4). Notably, the MED19a-*ORE1* complex is dissociated by root-specific *ELF18*-induced long non-coding RNA 1 (ELENA1), which propagates root nitrogen deficiency signals to shoots, mitigating leaf senescence (Cheng et al. 2023). The stability of ORE1 is further controlled post-translationally: the E3 ubiquitin ligase NLA (NITROGEN LIMITATION ADAPTATION) uses E2 conjugase PHO2/UBC24 (UBIQUITIN-CONJUGATING ENZYME 24) to polyubiquitinate ORE1 in the nucleus, functioning upstream of ORE1 (Park et al. 2018).

Hormones integrate closely with nitrogen and carbon starvation induced leaf senescence process. Apple *NAC* transcription factor *MdNAC4* (*NAC domain-containing protein 4*) responds to ABA, a senescence-promoting hormone, and directly binds the promoter of *MdSAG39* to enhance its expression, amplifying nitrogen deficiency-induced senescence (Wen et al. 2023). *WRKY1* (*WRKY DNA-BINDING PROTEIN 1*), another senescence regulator, is upregulated under nitrogen deficiency, and *WRKY1* induces the expression of SA biosynthesis genes, linking SA signaling to nitrogen response and activates nitrogen assimilation/transport genes, facilitating nitrogen remobilization while promoting senescence (Zhang et al. 2024a). This crosstalk aligns with broader senescence regulation, as SA and ABA often synergize to accelerate stress-induced senescence, while counteracting BR-mediated senescence delay.

For nitrate starvation, a special form of nitrogen stress, *A. thaliana* employs a root-shoot coordination pathway that interacts with hormone signaling. In the bud, nitrate starvation leads to senescence, but *nitrate transporter 1.5* (*NRT1.5*) senses nitrate deficiency through root function and regulates the absorption/transport of potassium (K^+^) to maintain the K^+^ level in leaves, thereby alleviating this senescence (Meng et al. 2016). Notably, NRT1.5 function overlaps with ethylene signaling, as ethylene upregulation under nitrate starvation can enhance NRT1.5-mediated K^+^ retention, balancing senescence and stress tolerance (consistent with ethylene’s dual role in senescence regulation). The miRNA maturation factor HASTY (HST) acts as a negative regulator. Nitrate starvation disrupts HST’s interaction with DCL1 and RAN1, altering miRNA dynamics (Sakuraba et al. 2024), and this process is linked to JA signaling, as JA-induced miRNAs (e.g. miR319) target senescence-related transcription factors, and HST dysfunction may perturb JA-miRNA crosstalk to accelerate senescence.

Other regulators further integrate nitrate starvation and hormone responses. *BBX14* (a BBX-type transcription factor) delays senescence under nitrate starvation and darkness, while its inhibition via artificial miRNA accelerates senescence (Buelbuel et al. 2023), and BBX14 has been shown to interact with ABA-responsive factors (e.g. ABF3) to fine-tune stress-senescence tradeoffs (Fig. 4). Additionally, the C3H-type zinc finger protein GDS1 (Growth, Development and Splicing 1) is ubiquitinated by the Anaphase Promoting Complex/Cyclosome (APC/C) under nitrate starvation, and GDS1 degradation relieves repression of senescence-promoting factors *PIF4/PIF5* (*PIGMENT INTERACTION FACTOR 4/5*), triggering premature senescence (Fan et al. 2023), and *PIF4/PIF5* also mediate ethylene-JA crosstalk, further linking nitrate starvation signaling to hormone-regulated senescence cascades.

Sugar partitioning, signaling, and utilization affect leaf source-sink regulated senescence (Kumar et al. 2019). Both sugar starvation and overaccumulation stimulate leaf senescence. Sugars serve as essential energy sources for biological processes and act as key cellular signaling metabolites that initiate and modulate leaf senescence (Kim et al. 2019; Asim et al. 2023). This regulatory function is particularly critical in annual plants, where sugar signaling not only optimizes growth and development but also accelerates the life cycle to ensure reproductive fitness (Wingler et al. 2022). Sugars function as essential nutrients for plants, their deficiency triggers adverse regulatory mechanisms that prematurely impair plant growth, including early leaf senescence. In rice, the sugar transporter *OsSWEET1b* (*Bidirectional sugar transporter SWEET1b*) functions as a positive regulator of leaf senescence by facilitating sugar translocation throughout the plant, a process that directly promotes senescence progression. Loss function of *OsSWEET1b* leads to sugar starvation, reduced photosynthesis, and leaf senescence (Zhang et al. 2024a). Counteracting this effect is *OsWRKY53*, a WRKY family transcription factor specifically activated during leaf senescence. OsWRKY53 exerts a dual inhibitory effect on *OsSWEET1b*. It suppresses *OsSWEET1b* gene expression and impairs its intracellular sugar transport activity. This dual inhibition reduces glucose accumulation in the cytoplasm of rice leaf cells, and the resulting perturbation of sugar homeostasis further amplifies senescence signals, ultimately driving leaf senescence (Chen et al. 2024) (Fig. 4).

Sugar overaccumulation also stimulates leaf senescence (Wingler et al. 2008). In wheat, long-term high light stimulates sugar overaccumulation and promotes leaf senescence. When exposed in high light, the content of fructose, sucrose, and starch increased dramatically, while chlorophyll binding proteins decreased substantially (Li et al. 2019). Notably, HXK1 (hexokinase 1), the first enzyme in hexose metabolism and acts as a pivotal sensor of sugar signaling, is involved in sugar overaccumulation induced leaf senescence. Overexpression of *HXK1* leads to high-glucose conditions and ROS accumulation. This process not only reduces photosynthetic efficiency but also triggers premature programmed cell death (PCD), thereby directly contributing to the onset of senescence (Zheng et al. 2021). Similarly, overexpression of *AtHXK1* in tomato affects sugar signaling, and leads to reduced photosynthesis, and rapid leaf senescence (Dai et al. 1999).​ Moreover, plastic mulching, a widely adopted agronomic practice, can upregulate the expression of *GhSUS* (*sucrose synthase*) and *GhINV* (*invertase*), causing enhanced accumulation of sucrose by 35.4%, glucose by 34.2%, fructose by 44.3%, and H_2_O_2_ by 12.4%. This synergistic increase collectively fuels ROS production, thereby accelerating premature leaf senescence (Qi et al. 2025).

### Salt and alkali affect leaf senescence

Soil salinization and alkalization impair plant growth and reduce crop yields, with salt stress emerging as a major environmental constraint that disrupts plant development and triggers early leaf senescence. To cope with salt stress, plants deploy adaptive strategies including regulating ion homeostasis, activating osmotic stress response pathways, modulating plant hormone signaling, and adjusting cytoskeletal dynamics and cell wall composition (Zhao et al. 2021; Wen et al. 2022; Ndecky et al. 2023). Notably, saline-alkali stress concurrently induces the expression of multiple stress-responsive genes, among which *SAGs* play pivotal roles. The salt-responsive gene *ORE1/ANAC092* acts as a master regulator of senescence. In *ore1* mutants, chlorophyll degradation is delayed under saline conditions, thereby postponing salt-induced leaf senescence (Balazadeh et al. 2010). Another key *SAG*, *SAG29*, which encodes a plasma membrane protein in *A. thaliana*, is upregulated under high salt stress. During senescence and stress responses, *SAG29* modulates cell viability by maintaining membrane integrity, a function critical for mediating salt-induced senescence (Seo et al. 2011).

Salt stress-induced leaf senescence is also tightly linked to JA signaling and PCD. In rice (*Oryza sativa*), the JA-deficient *aoc* mutant accumulates higher levels of Na^+^ in roots but lower Na^+^ in leaves, a phenotype associated with the suppression of HAK4-mediated Na^+^ transport in roots. Furthermore, leaves of the *aoc* mutant exhibit enhanced ROS scavenging capacity, reduced accumulation of senescence markers, and delayed chlorophyll degradation, collectively highlighting the distinct role of JA signaling in regulating salt-induced leaf senescence in rice (Ndecky et al. 2023). In *Reaumuria trigyna*, salt stress accelerates endogenous JA biosynthesis and upregulates the expression of *RtNAC100* (*NAC domain-containing protein 100*). Overexpression of *RtNAC100* activates the transcription of a ROS-producing gene *RtRbohE* (*Respiratory Burst Oxidase Homologue E*) and senescence marker genes *RtSAG12/20*, exacerbating salt-induced PCD by promoting ROS and Na^+^ accumulation. This cascade further activates central signaling pathways mediated by ROS and calcium ions (Ca^2+^), upregulates PCD-related genes, and ultimately drives salt-induced leaf senescence (Ma et al. 2021).

### High temperature affects leaf yellowing and senescence

High temperature, like salt and nitrogen deficiency, represents a key environmental stressor that induces premature leaf senescence, severely impairing plant growth and crop yields (Bulle et al. 2024). The mechanism of heat-induced senescence initiates with prolonged exposure to elevated temperatures, which disrupts cellular metabolism. This imbalance drives excessive accumulation of ROS while reducing the activity of antioxidant enzymes. The resulting ROS overload causes oxidative damage to chloroplasts, mitochondria, and other organelles, and further activates PCD, a cascade that directly triggers leaf senescence and cell death (Lee et al. 2016; Zhang et al. 2019; Li et al. 2021; Mittler et al. 2022; Qu et al. 2023).

Chloroplasts play dual roles in this process. They act as critical hubs for metabolic intermediates and primary sensors of high-temperature signals (Mittler et al. 2022; Zhang et al. 2022a). Chlorophyll, localized within chloroplasts, is essential for light absorption and energy conversion, maintaining high chlorophyll content typically mitigates photoinhibition and supports plant growth. However, when temperatures exceed the plant’s tolerance threshold, heat stress drives significant chlorophyll degradation, one of the most prominent phenotypic markers of leaf senescence. This heat-induced chlorophyll loss (e.g. heat-triggered leaf senescence) is accompanied by downregulation of photosynthesis-related genes, which reduces the activity of photosystem enzymes, impairs photosynthetic efficiency, and ultimately leads to growth retardation and yield reduction (Chen et al. 2014; Rehman et al. 2016). Thus, the ability to retain chlorophyll under heat stress directly correlates with enhanced high-temperature tolerance in plants.

A suite of regulatory factors fine-tunes heat-induced leaf senescence, with crosstalk to hormone signaling pathways, consistent with hormone-mediated stress responses in salt or nitrogen deficiency. In *A. thaliana*, NORE1/SAUL1 (Senescence-Associated E3 Ubiquitin Ligase 1) functions as a negative regulator of heat-induced senescence signaling, and it also modulates light intensity responses and the PHYTOALEXIN DEFICIENT 4 (PAD4) pathway, and its senescence- and cell death-regulating effects depend on downstream components like SGT1b (Suppressor of the G2 Allele of SKP1b), a positive regulator of R protein-mediated disease resistance (Lee et al. 2016). Deg2 (Degradation of periplasmic proteins 2), a serine protease localized to the stromal periphery of thylakoid membranes, is essential for leaf development and participates in heat-induced senescence: it inhibits leaf area expansion, alters chloroplast ultrastructure in older leaves, and attenuates the response of the light-harvesting protein Lhcb6 under heat stress, while repressing Deg2 delays senescence onset (Luciński et al. 2011). Additionally, PWL1 (a G-type lectin receptor-like kinase) positively regulates both leaf senescence and heat tolerance by maintaining ROS homeostasis and modulating PCD. Mutations in PWL1 impair downstream signaling activation, leading to ROS accumulation, abnormal chloroplast development, and premature senescence (Xu et al. 2023). Notably, these regulatory cascades often intersect with hormone signals, for example, JA may exacerbate heat-induced chlorophyll degradation by enhancing ROS accumulation, while ABA could fine-tune senescence via NORE1/SAUL1-related pathways, mirroring JA/ABA’s roles in other stress-induced senescence processes.

## Biological stress factors induced leaf yellowing and senescence

### Pathogen affects leaf yellowing and senescence

Pathogen infection triggers plant immune responses that engage in a dynamic interplay with leaf senescence: activated immunity can either delay senescence to preserve host fitness or accelerate it when overactivated, as unchecked defense responses drain resources and disrupt cellular homeostasis. Negative regulators of immunity are therefore critical for plant adaptability and productivity, as they suppress inappropriate defense activation, preventing premature leaf senescence (Breeze et al. 2011). Transcriptomic analyses have further highlighted the molecular overlap between these processes, revealing shared gene expression dynamics during senescence and pathogen invasion, and this has enabled the identification of numerous genes and signaling molecules such as ethylene that bridge immune responses and senescence regulation (Cristina et al. 2010; Breeze et al. 2011; Windram et al. 2012; Koyama et al. 2013; Yang et al. 2020; Zhang et al. 2022a; Chen et al. 2023).

Among the hormones mediating pathogen-senescence crosstalk, SA stands out as a central player: it is not only indispensable for plant defense against pathogens but also tightly linked to pathogen-induced leaf senescence (Zhang et al. 2013; Woo et al. 2019; Ding et al. 2020). SA is a well-characterized senescence inducer-senescent leaves consistently exhibit higher SA levels than non-senescent counterparts. Plant SA biosynthesis during pathogen infection primarily relies on the chloroplast-localized isochorismate (*ICS*) pathway, which involves two isochorismate synthase genes, *ICS1* and *ICS2* (Wildermuth et al. 2001; Garcion et al. 2008; Peng et al. 2021), while the phenylalanine ammonia lyase (PAL) pathway also contributes significantly to SA production under certain pathogenic stresses (Wildermuth et al. 2001; Peng et al. 2021; Ullah et al. 2023).

**Fig. 5:**
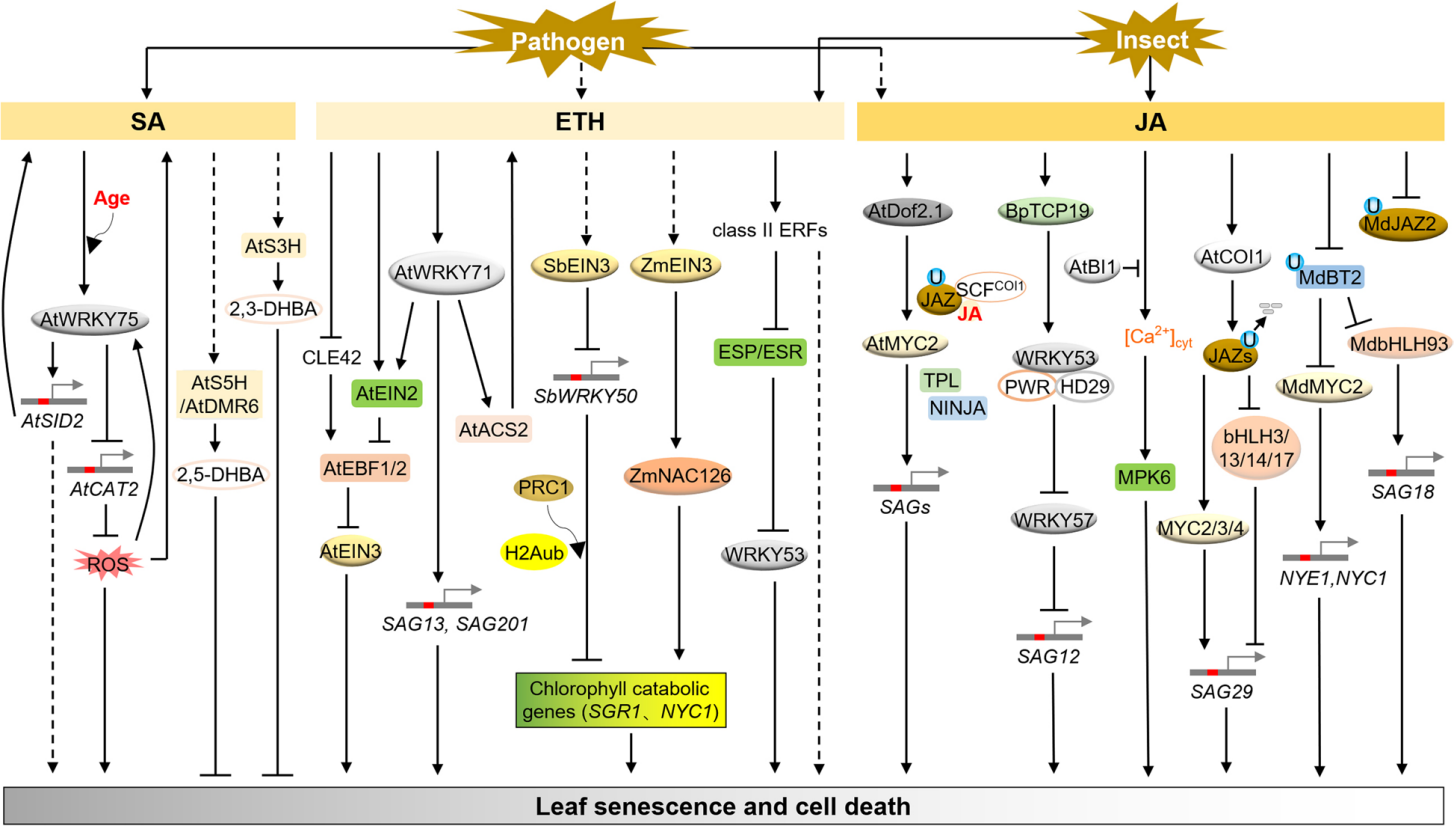
Biotic stress-induced leaf senescence signaling and regulatory network integrated by SA, ETH and JA. ETH, ethylene. JA, jasmonic acid. PRC1, Polycomb repressive complex 1

SA-mediated regulation of pathogen-induced senescence involves precise transcriptional and epigenetic control. In *A. thaliana*, the transcription factor WRKY75-previously linked to nitrogen deficiency-induced senescence-drives a tripartite amplification loop integrating SA, ROS, and senescence. WRKY75 promotes SA biosynthesis by upregulating the expression of *SA INDUCTION-DEFICIENT2* (*SID2*, encoding ICS1) and enhances ROS accumulation by repressing the expression of *CATALASE2* (*CAT2*), creating a positive feedback loop that accelerates senescence (Guo et al. 2017) (Fig. 5). SA levels must be tightly balanced: insufficient SA impairs defense against pathogens and abiotic stresses, while excessive SA reduces plant fitness-inducing dwarfism or premature senescence. The *DMR6/S5H* (*Downy Mildew Resistant 6*) and *DLO1/S3H* (*DMR6-LIKE OXYGENASEs 1*) genes serve as critical regulators balancing SA-mediated leaf senescence and plant immune defense, functioning as key SA-responsive factors with dual roles in promoting senescence and inhibiting immune responses (Zhang et al. 2017a). Both genes encode 2-oxoglutarate iron II-dependent oxygenase but exhibit distinct expression patterns and catalytic activities throughout plant development: *DMR6/S5H* is active from the seedling stage to senescence, and in *A*. *thaliana*, it specifically hydroxylates SA at the C5 position to catalyze the formation of 2,5-dihydroxybenzoic acid (2,5-DHBA), thereby modulating SA-dependent signaling pathways. In contrast, *DLO1/S3H* displays a more specific expression profile, being predominantly induced in mature and senescent leaves, where it converts SA into 2,3-dihydroxybenzoic acid (2,3-DHBA) through C3 hydroxylation. This process promotes SA catabolism, ultimately attenuating leaf yellowing during senescence. Together, these two enzymes fine-tune SA homeostasis through differential metabolic modification, playing indispensable roles in maintaining the dynamic balance between leaf senescence progression and plant immune system regulation (Van et al. 2020; Chan et al. 2022; Van et al. 2025).

Beyond SA, mitogen-activated protein kinase phosphatases (MKPs) and calcium-dependent protein kinases (CPKs/CDPKs) integrate pathogen-induced immune signaling with leaf senescence, complementing hormone-mediated regulation. MKPs (e.g. MKP1, MKP2)-monomeric bispecific phosphatases-modulate the activation of MAP kinases MPK3 and MPK6, key transducers of pathogen stress signals, thereby fine-tuning immune responses to avoid senescence overactivation (Bartels et al. 2009; Jiang et al. 2017). CPKs further link calcium signaling (a rapid immune response) to senescence: in *A. thaliana*, CPK1 (Calcium-dependent Protein Kinase 1) phosphorylates ORE1, a master senescence regulator previously identified in nitrogen deficiency and salt stress responses-at an intrinsically disordered region. This phosphorylation enhances ORE1’s transcriptional activation of *BIFUNCTIONAL NUCLEASE1* (*BFN1*, a senescence-associated nuclease), directly connecting pathogen-induced calcium signaling to senescence execution (Durian et al. 2020).

### Insect pests affect leaf senescence

Insect herbivory is a major biotic stress that induces leaf senescence in plants, primarily via nutrient exploitation and stress signal activation-with plant hormones (notably JA, ethylene, and SA) serving as core regulatory hubs. This process is conserved across species but exhibits species-specific molecular mechanisms (Yue et al. 2012; Zhuo et al. 2020; An et al. 2021; Pang et al. 2024).

In *A. thaliana*, P-Type II ATPases ACA10 (Autoinhibited Ca^2+^ ATPase 10) and ACA12 (Autoinhibited Ca^2+^ ATPase 12) mediate resistance to herbivory-induced senescence. The *aca10 aca12* double mutant shows accelerated senescence (petiole chlorosis spreading to leaf laminas) after feeding by *Spodoptera littoralis* caterpillars or aphids, accompanied by reduced chlorophyll, increased fructose, and elevated amino acids-confirming herbivory-driven senescence (Fotouhi et al. 2022). In *Medicago truncatula*, ethylene is critical for aphid-induced senescence. Wild-type A17 plants undergo senescence after infestation by *Aphis kondoi*, *Therioaphis trifolii*, or *A. pisum*, while the ethylene-insensitive sickle mutant avoids senescence (even with high aphid density) and retains more biomass, highlighting ethylene’s non-redundant role (Zhang et al. 2020). For *Terminalia catappa*, beetle herbivory alters leaf chemistry to promote senescence. Senesced damaged leaves show 44% lamina loss, reduced lignin (39%) and nitrogen (22%), and altered nutrient stoichiometry-linking herbivory to senescence-associated nutrient remobilization (Marler et al. 2018).

High-density psyllid infestation induces chlorosis (reduced chlorophyll, carotenoids, anthocyanins) mimicking natural senescence, though uniquely delaying necrosis to prevent plant dehydration. In basil, aphid or thrips feeding activates the senescence-specific pheophorbide a oxygenase/phyllobilin (PAO/PB) pathway: infested regions accumulate novel phyllobilins (Ob-NCC-40, Ob-YCC-45) at higher levels than healthy tissue, directly linking herbivory to senescence-associated chlorophyll catabolism (Steinbauer et al. 2014).

JA, a key anti-herbivore hormone, fine-tunes herbivory-senescence crosstalk. The salivary protein Rp2155 of Chilo partellus (upregulated during feeding) exacerbates soybean dieback by disrupting the SA-JA signaling balance-knockout of Rp2155 alleviates senescence (Guo et al. 2014; Arnao et al. 2018). In apple, the MdWRKY75-MdVQ10 (VQ motif-containing protein 10) module drives damage-induced senescence (Zhang et al. 2023). This aligns with JA’s conserved function in stress-induced senescence (e.g. salt, high temperature), where it balances defense and growth to maintain plant fitness.

## Make sense of leaf senescence via synthetic biology breeding

**Fig. 6:**
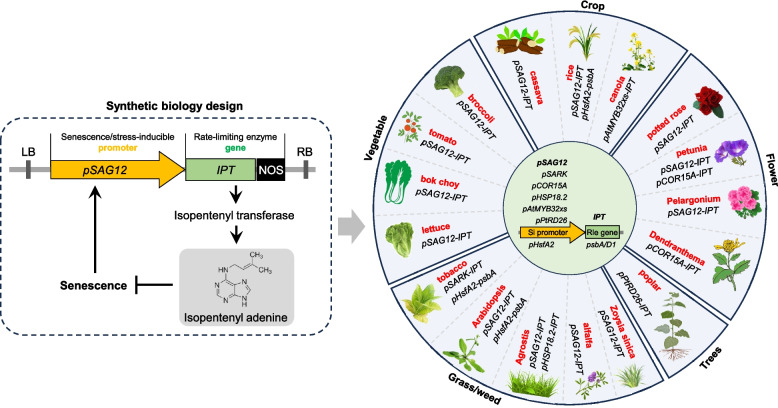
Application of stress-induced leaf senescence. The synthetic biology model of *pSAG12-IPT* was introduced and modified in multiple plants including crops, vegetables, grass/weed, flowers and tree species

By employing genetic toggle switches and repressilators, synthetic biology enables living systems to exhibit complex, electronics-inspired behaviors (Benner et al. 2005; Khalil et al. 2010). The *P*_*SAG12*_*-IPT* system exemplifies the earliest synthetic biological designs in plants, integrating *P*_*SAG12*_, a promoter specific to senescence, with the *isopentenyltransferase* (*IPT*) gene to create a self-regulatory mechanism that inhibits senescence, induces cytokinin synthesis through self-feedback, and specifically delays leaf senescence without impacting the development of other parts of the plants (Gan et al. 1995; Gan et al. 1997) (Fig. 6, Table 1). By implementing strategic control over leaf senescence using the *P*_*SAG12*_*-IPT* or similar systems, the delayed developmental senescence phase has been significantly extended in many species, and some plants show stress-tolerant phenotype (Guo et al. 2014).

**Table 1:** Summary of the role of genes related to leaf senescence in molecular breeding

Species	leaf senescence gene	Promoter	Type of enhanced phenotype	Defects or limitations	Reference
Poplar	*IPT, PtRD26*	*p_PtRD26 promoter*	Improved growth, enhanced drought tolerance	No obvious abnormalities	(Wang et al. 2022a, b)
Arabidopsis, tobacco, rice	*D1 gene*	*p_HsfA2*	Heat tolerance, enhanced photosynthetic capacity, growth promotion, increased biomass, and improved grain yield	Applicability not verified in non-model plants and stability and ecological safety under long-term not clarified	(Chen et al. 2020a, b)
Miniature potted rose	*IPT*	*p_SAG12*	Ethylene resistance, The leaves remain green	Insufficient repeatability	(Zakizadeh et al. 2013)
Rice	*OsNAP*	*CRES-T*	Grain yield per plant increased by 24%, spikelet fertility rate increased by 6%	No obvious abnormalities	(Tang et al. 2012)
Maize	*ZmNAP*	*RNAi*	Stay-green phenotype, 1000-grain weight increased by 15–30%	No obvious abnormalities	(Zhang et al. 2012)
Cotton	*IPT*	*PGhcysp*	Improved salt tolerance and significant increase in lint yield	No relevant negative reports	(Liu et al. 2012)
Rice	*IPT*	*p_SARK*	Significantly improved grain yield under water stress, protection of photosynthetic protein complexes	No relevant negative reports	(Peleg et al. 2011)
Creeping bentgrass	*IPT*	*p_SAG12*	Improved turf quality under drought stress, maintained photosynthetic activity, increased root viability, biomass accumulation increased	No relevant negative reports	(Merewitz et al. 2011)
Peanut	*IPT*	*p_SARK*	Peanut fruit yield increased by 58% under water-limiting conditions	No yield increase under well-watered conditions	(Qin et al. 2011)
Creeping bentgrass	*IPT*	*p_SAG12*	Drought tolerance, Root-crown ratio	The genetic stability needs to be improved and the phenotype is not obvious	(Merewitz et al. 2010)
Creeping bentgrass	*IPT*	*pHSP18.2*	Drought tolerance, Root-crown ratio	The phenotype is not obvious and the mechanism of oxidation regulation is not clear	(Merewitz et al. 2010)
Cassava	*IPT*	*p_SAG12*	Drought tolerance, biomass	Some phenotypic differences are not obvious	(Zhang et al. 2010)
Tobacco	*IPT*	*p_SARK*	Enhanced resistance to wilting	No obvious abnormalities	(Rivero et al. 2010)
Creeping bentgrass	*IPT*	*p_SAG12*	Heat tolerance, tillering number, root biomass and total root length	The genetic stability needs to be improved	(Xu et al. 2009)
Wheat	*IPT*	*p_SAG12*	Delayed leaf senescence, enhanced nitrate uptake, increased nitrate reductase activity	No yield increase	(Sykorova et al. 2008)
Tobacco	*IPT*	*p_SARK*	Drought tolerance, biomass	No obvious abnormalities	(Rivero et al. 2007)
Carnation	*DHS*	*Antisense DHS*	Delayed petal senescence	No relevant negative reports	(Hopkins et al. 2007)
Tomato	*IPT*	*p_SAG13*	Biomass	Phenotypic weakening	(Swartzberg et al. 2006)
Wheat	*NAP homologs*	*RNAi*	Flag leaf senescence delayed by 24 days	Grain protein, zinc, and iron contents decreased by more than 30%, reduced nutrient translocation	(Uauy et al. 2006)
Alfalfa	*IPT*	*p_SAG12*	Blade life	Poor consistency	(Calderini et al. 2007)
Arabidopsis	*AtNAP*	*T-DNA insertion*	Leaf senescence delayed by up to 10 days; delay degree negatively correlated with AtNAP transcript level	No obvious abnormalities	(Guo et al. 2006)
Tomato	*DHS*	*Antisense DHS 3' untranslated region*	Delayed post-harvest softening and senescence, reduced polygalacturonase activity, delayed electrolyte leakage	Male sterility in one transgenic line	(Wang et al. 2005)
Chrysanthemum	*IPT*	*p_COR15a*	The leaves remain green	Aging delay is reversible	(Khodakovskaya et al. 2005)
Arabidopsis	*IPT*	*p_SAG12*	Waterlogging tolerance, biomass	Affects the normal regulation of stomata in crops	(Huynh et al. 2005)
Petunia	*IPT*	*p_COR15a*	The ability to retain green in the dark after cold induction	Insufficient repeatability	(Khodakovskaya et al. 2005)
Maize	*IPT*	*SEE1*	Delayed leaf senescence, reversal of pistil abortion during flower development	No relevant negative reports	(Robson et al. 2004)
Petunia	*IPT*	*p_SAG12*	Delayed corolla aging	Line-specific morphological abnormalities	(Chang et al. 2003)
Tomato	*IPT*	*p_SAG12*	Leaf senescence delayed by 1 week, increased fruit setting, yield increased by 20%	No obvious abnormalities	(Feng et al. 2003)
Rice	*IPT*	*p_SAG12*	Biomass and plant height decrease	No obvious developmental abnormalities	(Lin et al. 2002)
Broccoli	*IPT*	*p_SAG12*	no	Low conversion efficiency	(Gapper et al. 2002)
Rapeseed	*IPT*	*p_SAG12*	Yield increased by 6.4%−32.9%	No obvious abnormalities	(Luo et al. 2002)
Lettuce	*IPT*	*p_SAG12*	Chlorophyll retention in harvested heads for 7 days, no yellowing of basal leaves	No obvious abnormalities	(McCabe et al. 2001)
Tobacco	*IPT*	*p_SAG12*	Leaf morphology, canopy structure	The nitrogen utilization efficiency in the field has not improved	(Jordi et al. 2000)
Broccoli	*IPT*	*p_SAG12*	Delayed post-harvest yellowing, chlorophyll retention in leaves and florets	No relevant negative reports	(Chen et al. 2001)
Arabidopsis	*IPT*	*p_SAG12*	Biomass increased by 35%−85% under waterlogging conditions, waterlogging tolerance enhanced	No obvious abnormalities	(Zhang et al. 2000)
Tobacco	*IPT*	*p_SAG12*	Flowers produced, biomass, seed yield, plant height, leaf number	Slightly lower chlorophyll content in young leaves under insufficient nutrient supply	(Gan et al. 1995)

Leaf senescence is a crucial aspect of plant development, exerting considerable influence on plant yield, biomass accumulation, and nutritional quality. The senescence process of plant leaves requires the coordinated regulation of multiple *SAGs*, with *SAG12* being the most widely utilized reference gene for the characterization of leaf senescence. As the life cycle of leaves concludes, an increase in the transcript level of *SAG12* has been observed (James et al. 2018). Investigations into the physiological and agronomic traits of cassava have revealed that the induced expression of the *IPT* gene affects photosynthesis, sugar allocation, and nitrogen distribution within the plant (Zhang et al. 2010). Numerous studies have consistently confirmed the presence and functionality of the *P*_*SAG12*_*-IPT *(*pSAG12-IPT*) system in agricultural, horticultural and forestry plant species, including *A. thaliana* (Huynh et al. 2005), creeping bentgrass (Xu et al. 2009), tomato (Swartzberg et al. 2006), zoysia grass (Zhang et al. 2005), cassava (Zhang et al. 2010), potted roses (Zakizadeh et al. 2013), morning glory (Chang et al. 2003), lettuce (McCabe et al. 2001), rice (Lin et al. 2002), broccoli (Gapper et al. 2002), tobacco (Jordi et al. 2000), and alfalfa (Calderini et al. 2007). Moreover, other genes with induced expression, such as *pHsfA2-psbA* (Chen et al. 2020a, b), *pAtMYB32xs-IPT* (Kant et al. 2015), *pCOR15A-IPT* (Khodakovskaya et al. 2005), *pPtRD26-IPT* (Wang et al. 2022a, b), *Phsp18.2-IPT* (Xu et al. 2016), and *pSARK-IPT* (Rivero et al. 2007) (Table 1), can similarly influence leaf senescence process and enhance plant tolerance to multiple stresses. For example, transgenic petunia expressing *pCOR15A-IPT* maintained a normal phenotype following black cooling treatment, showing no signs of leaf wilting (Khodakovskaya et al. 2005). Additionally, *pSARK-IPT* design enhances drought resistance through cytokinin induction, exhibiting reduced susceptibility to photosynthesis under drought conditions and demonstrating a more pronounced effect on leaf greenness retention when comparing transgenic and wild-type phenotypes (Rivero et al. 2007). Excessive synthesis of cytokinin through the expression of the IPT has been identified as a primary strategy to mitigate the senescence process in transgenic plants (Gan et al. 1996; Li et al. 2000; Guo et al. 2008; Rivero et al. 2010). The direct regulation of IPT (*P*_*SAG12*_*-IPT*) by the promoter of senescence-specific genes, such as *SAG12*, to establish a self-regulating cytokinin production system is particularly advantageous for practical applications and is steadily advancing toward commercial viability. Additionally, methods for regulating senescence that involve specific age-related transcription factors, including NAP and the translation initiation factor eIF-5A, also demonstrate promising prospects with considerable potential for commercialization (Guo et al. 2014).

Although *IPT*-mediated systems represented by *pSAG12-IPT* have achieved delayed senescence and enhanced stress resistance in multiple species. Their application still has limitations, such as long-term field observations have found that the imbalance of nutrient distribution in transgenic plants may affect normal growth in some species. In response to these limitations, at the metabolic level antioxidant enzymes such as SOD and APX (Ascorbate Peroxidase) can enhance the ability to clear ROS, and when used in combination with *IPT*, they can significantly improve the drought stress resistance of creeping bentgrass and make up for the stress resistance deficiency of the single *IPT* strategy. Emerging synthetic biology methods have further expanded the regulatory dimension. Precise editing of the *SAG12* promoter mediated by CRISPR-Cas9 can achieve senescence-specific spatiotemporal regulation without the need to transfer exogenous genes. Synthetic transcription factors such as dCas9-VP64 can modularize the expression of *SAGs* and avoid the problem of CTK excess.

## Future prospect of stress-induced leaf senescence

The process of senescence in plants encompasses two dimensions. On one hand, it results in the apoptosis of cells and a significant reduction in the number of chloroplasts, which adversely affects photosynthesis. On the other hand, the senescence of older or stressed leaves contributes essential nutrients to younger or developing tissues, thereby enhancing the plant's overall survival. Numerous genes associated with the senescence process in leaves have been identified and examined to deepen our understanding of leaf senescence. However, some critical compounds remain unidentified, indicating a need for further investigation and exploration of these regulators such as splicing factors (SFs). The advancement of omics technologies has enabled the flexible application of various experimental designs, including transcriptomics, proteomics, metabolomics, genomics, and epigenomics (Kim et al. 2016). The regulation of a single gene is overly simplistic, as genes typically function within a complex network during leaf senescence process. Therefore, a more holistic approach that involves the simultaneous knockout or overexpression of multiple interconnected genes may be necessary to investigate the integrated regulatory network of these genes. Moreover, the interdisciplinary field that encompasses both animal and plant studies has attracted increasing attention, and this raises the challenges of whether insights gained from animal drug screening can be applied to elucidate the anti-senescence mechanisms in plant leaves. Additionally, it prompts inquiry into the potential for extrapolating the design of regulatory hormone or network systems in plant leaves to animal studies (Wang et al. 2023a).

The primary aim of contemporary plant breeding is to increase plant yield through the implementation of advanced technologies or designs (Gao et al. 2021; Fu et al. 2025). The primary aim of study on plant senescence extends beyond merely understanding this complex phenomenon, and it also seeks to leverage foundational discoveries to develop techniques for regulating senescence in plant practices, and a significant strategy in this pursuit can be operated by altering leaf senescence process. Through continuous data collection and iterative training, artificial intelligence (AI) can automatically uncover hidden patterns in massive, high-dimensional, multimodal biological data, driving a paradigm shift from merely describing biological phenomena to predicting molecular functions, and ultimately enabling the rational design of biomolecules, thereby enabling the targeted modification of cis-acting elements at the genetic level (Harfouche et al. 2019; Farooq et al. 2024; Fu et al. 2025), including leaf senescence associated cis-acting elements. This capability can augment or suppress functional domains, ultimately leading to improved phenotypic outcomes. The rapid evolution of AI has introduced new challenges and avenues for research in the field of leaf senescence. Machine learning (ML) enables iterative learning from patterns across diverse phenotypes of numerous labeled plants by a variety of algorithms, such as support vector machines (SVM), K-nearest neighbors (KNN), and random forests (RF). This capability facilitates the prediction of new and previously unobserved data following the training process. Techniques such as support vector regression (SVR), KNN, and partial least squares (PLS) can be employed to develop quantitative predictive models for sucrose spectra, utilizing the sucrose concentration at the end of tomato petioles as an indicator to assess leaf senescence, and this approach aids in the removal of senescent leaves, thereby enhancing the yield of greenhouse tomatoes (Ni et al. 2024). As a subset of ML, deep learning (DL) addresses the limitations associated with traditional ML methodologies, allowing for more accurate extraction of features and patterns in complex tasks (Bodner et al. 2018). Consequently, this has led to the development of various analytical methods, including genome-wide association studies (GWAS), multilayer perceptrons (MLP), convolutional neural networks (CNN), recurrent neural networks (RNN), and graph convolutional networks (GCN). Image-based trait analysis has been applied in leaf senescence study. A genome-wide association study, combined with image-based trait analysis and transcriptomic data, has established that OsMYB21 positively regulates dark-induced leaf senescence in both indica and japonica rice, while OsSUB1B negatively influences its progression (Li et al. 2025).

The primary aim of contemporary plant breeding is to increase plant yield through the implementation of advanced technologies or designs, and a significant strategy in this pursuit can be operated by altering the leaf senescence process (Schippers et al. 2015). Through continuous data collection and iterative training, AI can achieve enhanced accuracy in gene sequencing, thereby enabling the targeted modification of cis-acting elements at the genetic level, including leaf senescence associated cis-acting elements. This capability can augment or suppress functional domains, ultimately leading to improved phenotypic outcomes (Farooq et al. 2024). Additionally, advancements in synthetic biology have markedly enhanced molecular design breeding. Synthetic biology seeks to design and construct new biological systems or modify existing ones to achieve specific functions and applications (Benner et al. 2005). This progress encompasses the investigation of genetic elements, the design of gene circuits, and the optimization of metabolic pathways, all aimed at facilitating the intentional design and modification of plant leaf genes at the molecular level (Ausländer et al. 2017). For example, rice *OsLC1* (*Leaf Inclination 1*) regulates lignin and flavonoid biosynthesis by metabolomics analysis, which showed the molecular mechanism of the coordinated regulation of metabolic pathways and cell development (Wu et al. 2025). Consequently, breeding experiments can be conducted with greater precision and efficiency in alignment with specific human objectives. For example, the *pSAG12-IPT* gene has been engineered in various studies to delay leaf senescence without negatively impacting the development of other plant structures (Jordi et al. 2000; McCabe et al. 2001). Furthermore, this gene has demonstrated the ability to reduce leaf sensitivity to stress and mitigate stress-induced damage to leaves (Huynh et al. 2005). Recently, advancements in protein prediction technologies have further accelerated this progress (Jumper et al. 2021). Additionally, the application of AI can stimulate premature leaf senescence, thereby shortening the breeding cycle and conserving land resources. For instance, the *A. thaliana* transcription factor WRKY1 plays a crucial role in multiple key steps of leaf senescence. Overexpression of WRKY1 accelerates leaf senescence, promotes the efficient transfer of nutrients from leaves to seeds, and enables rapid breeding (Zhang et al. 2024b). Conversely, it can also delay leaf senescence, enhance photosynthesis, and improve plant resistance and yield. MFMGP (Machine Learning Fusion Model for Genomic Prediction) is a machine learning-based whole-genome selection tool. It optimizes prediction accuracy through DNNGP, XGBoost (Extreme Gradient Boosting), etc. and performs particularly well in the prediction of complex agronomic traits (stress resistance, yield traits) (Zhang et al. 2025). The applications of machine learning (ML) and deep learning (DL) are crucial in addressing issues related to plant leaf resistance and aging.

**Fig. 7:**
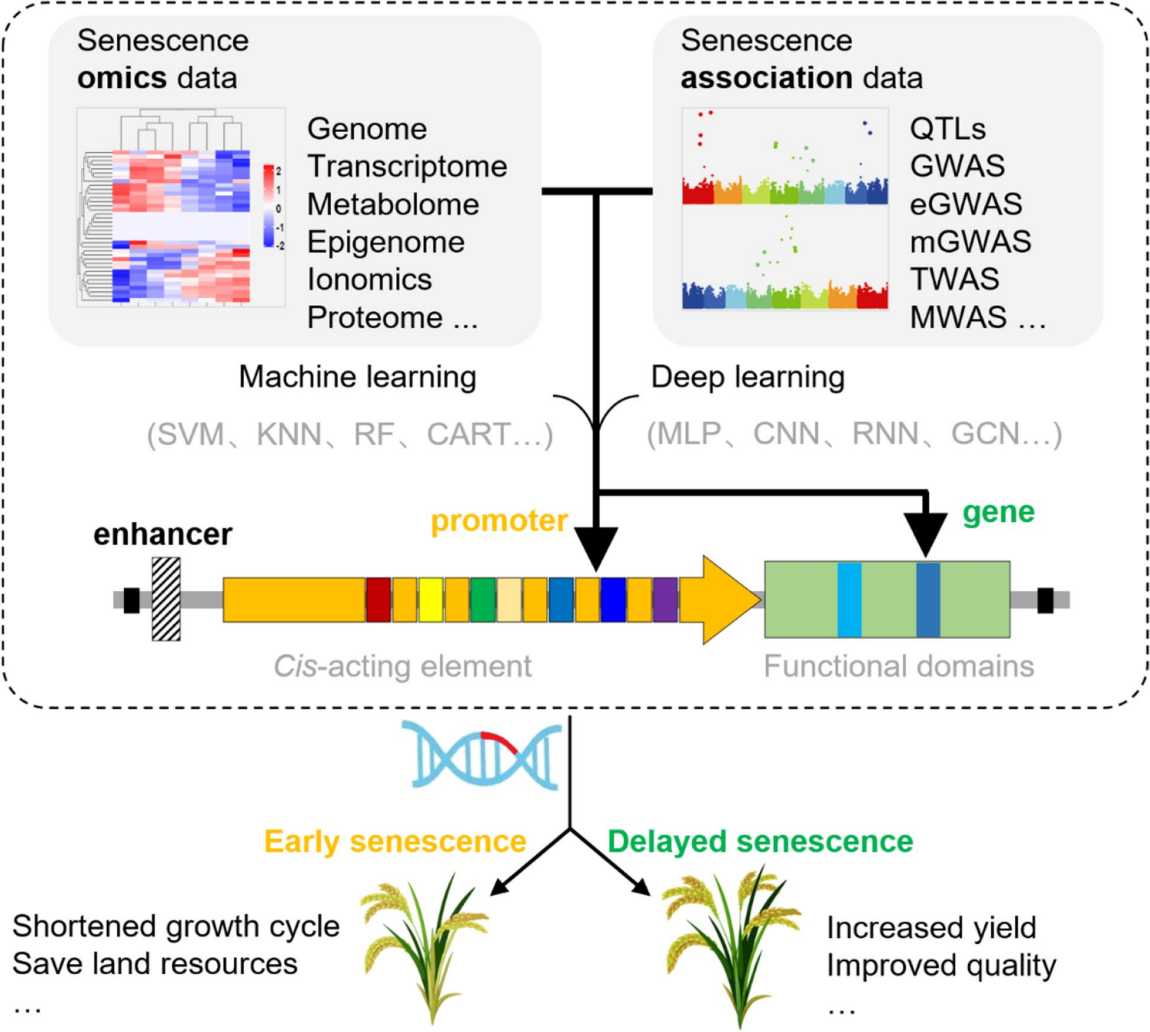
Applications of machine learning (ML) and deep learning (DL) in senescence/stress associated crop breeding. QTLs, quantitative trait loci; GWAS, genome-wide association study; eGWAS, expression GWAS; mGWAS, metabolome GWAS; TWAS, transcriptome-wide association study; MWAS, metabolome-wide association study; SVM, support vector machine; KNN, K-nearest neighbors algorithm; RF, random forest; CART, classification and regression tree; MLP, multilayer perceptron; CNN, convolutional neural network; RNN, recurrent neural network; GCN, graph convolutional network

The integration of synthetic biology and AI has great potential to promote plant science research, particularly in the area of plant breeding through the intelligent design of senescence-associated elements. By leveraging machine learning, data mining, and automated optimization capabilities inherent in AI, synthetic biology has achieved significant advancements in the systematic engineering of gene networks (transcriptional), non-coding regulatory RNAs (translational), and protein signal transduction circuits (post-translational). AI can be recognized as a fundamental tool for the design of both senescence- and stress-associated promoters, genes, and protein elements within the realm of synthetic biology (Fig. 7). Through data-driven approaches and algorithmic optimization, AI can markedly enhance the accuracy and functionality of biological components. For instance, the application of CNNs or transformer models, such as DNABERT (DNA Bidirectional Encoder Representations from Transformers), allows for the prediction of the strength and tissue specificity of conserved motifs, including TATA boxes and transcription factor binding sites within promoter sequences (Ji et al. 2021). Furthermore, AI can facilitate the generation of 5' untranslated regions (UTRs) or 3' UTRs to modulate mRNA stability and translation efficiency. AI models, such as long short-term memory networks (LSTMs) or graph neural networks (GNNs) (Yu et al. 2019; Wu et al. 2020), can learn the codon usage frequency of host cells and autonomously generate gene sequences that are highly expressed, thereby circumventing potential bottlenecks associated with transfer RNA (tRNA). Looking forward, the integration of AI with senescence-associated synthetic biology design holds significant promise for ensuring food security and in the near future.

## Data Availability

All data discussed in this review are sourced from the cited references. No additional data are associated with this article.

## Abbreviations

2,3-DHBA: 2,3-Dihydroxybenzoic acid
2,5-DHBA: 2,5-Dihydroxybenzoic acid
ABA: Abscisic acid
AI: Artificial intelligence
BR: Brassinosteroids
Ca^2+^: Calcium ions
CNN: Convolutional neural networks
COG: Conserved oligomeric Golgi
CTK: Cytokinins
DL: Deep learning
DNABERT: DNA Bidirectional Encoder Representations from Transformers
DNNGP: Deep Neural Network for Genomic Prediction
ETH: Ethylene
GCN: Graph convolutional networks
GNNs: Graph neural networks
GWAS: Genome-wide association studies
ICS: Isochorismate
INV: Invertase
IPT: Isopentenyltransferase
JA: Jasmonic acid
KNN: K-nearest neighbors
LSTMs: Long short-term memory networks
m^6^A: N^6^-methyladenosine
MeJA: Methyl jasmonate
MFMGP: Machine Learning Fusion Model for Genomic Prediction
miRNAs: MicroRNAs
miR775: MicroRNA 775
ML: Machine learning
MLP: Multilayer perceptrons
PCD: Premature programmed cell death
PLS: Partial least squares
RF: Random forests
RNN: Recurrent neural networks
ROS: Reactive oxygen species
SA: Salicylic acid
SVM: Support vector machines
SVR: Support vector regression
tRNA: Transfer RNA
UTRs: Untranslated regions
XGBoost: Extreme Gradient Boosting
